# The Comparison of Self-regulation and Affective Control in Methamphetamine and Narcotics Addicts and Non-Addicts

**DOI:** 10.5812/ijhrba.8442

**Published:** 2013-03-12

**Authors:** Kolthoum Tayyebi, Abbas Abolghasemi, Majid Mahmood Alilu, Nader Monirpoor

**Affiliations:** 1 Department of Clinical Psychology, Islamic Azad University, Ardabil Branch, Ardabil, IR Iran; 2 Department of Psychology, University of Mohaghegh Ardabili, Ardabil, IR Iran; 3 Department of Clinical Psychology, University of Tabriz, Tabriz, IR Iran; 4 Department of Clinical Psychology, Islamic Azad University, Qom Branch, Qom, IR Iran

**Keywords:** Narcotics, Methamphetamine, Self-regulation, Affective control

## Abstract

**Background:**

Increased prevalence and widespread use of methamphetamine is the public challenge and worry in the world. It seems that low levels of self-regulation and affective control to carry up probability of psychoactive drugs abuse.

**Objectives:**

The purpose of the present study is the comparison of self-regulation and affective control in methamphetamine and narcotics addicts and non-addicts.

**Materials and Methods:**

In this causative-comparative study, 80 addicts (40 methamphetamine addicts and 40 narcotic addicts) who referred to self-reference quitting addictive centers in Miyaneh, Iran, participated in convenience sampling. Then, they matched up with 40 non-addicts according to age, sex, educational level, and marital status. To collect data, we used self-regulation questionnaire and affective control scale. The data was analyzed by multivariate analysis of variance (MANOVA) and LSD test.

**Results:**

Result shows that there is a significant difference between methamphetamine addicts and narcotics addicts and non-addicts in self-regulation and affective control (P = 0.001).

**Conclusions:**

This finding indicates that low self-regulation and affective control is a risky factor in psychoactive drugs abuse.

## 1. Background

The abuse of methamphetamine has increased worldwide in recent years, and methamphetamine use is often associated with psychological disorders (1). Therefore, researchers believe that self-regulation and self-control (and Affective control) can be affected by substance addicts’ behaviors. Self-regulation, defined as the psych’s efforts to control its internal states, processes and functions for the purpose of achieving a higher goal (2). Self-regulation is also the capacity to override one’s thoughts, emotions, impulses, and automatic or habitual behaviors. People must constantly adapt and adjust their behavior to new environments and demands by self-regulating. Furthermore, self-regulation influences many of the major problems faced by people as individuals and society as a whole. For instance, poor self-regulation can be very difficult to abstain from drug and alcohol use after one has established a routine of regular use. Therefore, self-regulation can allow individuals to resist drug abuse, and thereby reduce various problems associated with such abuse (3, 4). Also, it seems that self-regulation holds individual’s behavior under the control of social standards. Behaviors that are rewarded in the society, can lead to the self-regulation (5). Therefore, when individuals use drugs, this rule is broken and drug use would reduce by addict's self-regulation (3). On the other hand, in every society, self-control and affective control is important for getting along with others. A person who cannot control his or her thoughts, feelings, or behavior is more likely to lash out in anger when frustrated, handle conflicts less constructively, engage in antisocial behavior (6); use drugs and commit substance-related problem behaviors presumably due to deficits in inhibitory control (7, 8). Copeland and Sorensen found that methamphetamine users were more likely to have a psychiatric diagnosis. Mood disorders accounted for 71% of the diagnoses among methamphetamine users (1). Sussman et al. have shown that behavioral self-control is inversely related to drug use, controlling for relatively unchangeable disorders of personality (9). Glassman et al. found that self-control strategies are associated with alcohol consumption and self-regulation is also related to alcohol problems (10). De wall et al. found that the capacity for self-control and self-regulation is a limited resource that operates like a strength or energy, and when this capacity is depleted, people are less successful in self-regulation and therefore they should be more likely to act aggressively if the aggressive impulse arises (11). Vik showed that there is a positive relation between female methamphetamine abusers and psychiatric disorders (anxiety disorder, depression and interpersonal sensitivity), so that 53% of methamphetamine abusers had criteria for an affective disorder, and 46% of they had criteria for an anxiety disorder. (12). Results of a study by Otten et al. showed that low levels of self-control are predictive of co-occurrence of cannabis use and depressive symptoms. Also, this study showed that individuals with low self-control may be incapable of inhibiting impulsivity which is a sign for substance use (13). Study results of Cole et al. showed that affect, cognitive and behavior indicators of self-regulation were significant predictors of substance use disorder (2).

## 2. Objectives

Nowadays, there is an increasing tendency among youth to use methamphetamine, and few research works have been conducted on the importance of the self-regulation and affective control skills of these groups; with emphasis on these findings, educational programs of self-regulation and affective control skills in therapeutic design in therapy centers could be applied. Therefore, the aim of this study is the comparison of self-regulation and affective control in methamphetamine and narcotics addicts and non-addicts.

## 3. Materials and Methods

### 3.1. Participants and Plan

In this causative-comparative study, 80 addicts (40 methamphetamine addicts and 40 narcotic addicts), who referred to self-reference quitting addictive centers in Miyaneh, Iran (2011), participated in the convenience sampling. Then, they were matched up with 40 non-addicts according to age, sex, educational level and marital status. The criteria of selected people included being: male, married, in the age range of 20 to 39 years, and literate. The criteria of exclusion were suffering from: psychotic disorders, bipolar or dissociative disorders; or a severe somatic disease.

### 3.2. Measures

### 3.2.1. The Self-Regulation Questionnaire (SRQ)

The questionnaire had 63 items; the subscales to measure the ability to develop, implement, and flexibility to maintain a planned behavior in order to achieve one's goals. Items were answered on a 5-point Likert scale with the following scale points (Strongly disagree, Disagree, Uncertain or Unsure, Agree and Strongly Agree). Scores above 239 indicate high (intact) self-regulation capacity; Scores of 214-238 indicate intermediate (moderate) self-regulation capacity, and scores less than 213 indicate low (impaired) self-regulation capacity. Reliability of the SRQ appears to be excellent. In a community sample of 83 people with varying levels of alcohol problem severity, the SRQ was administered twice, within 48 hours, to test the stability of scores it provides. Test-retest reliability for the total SRQ score was high (r = 0.94, P = 0.001). The SRQ has also shown strong convergent validity with concomitant measures. In community sample Aubrey et al. (1994), SRQ score was significantly and inversely correlated with volume of alcohol consumption per occasion (r = -0.23, P = 0.04) and with negative consequences of drinking (r = -0.46, P = 0.001). This means that people with lower scores on the SRQ were more likely to be heavy and problem drinkers (14). According to the Cronbach's alpha of this study for the whole scale, the reliability coefficient of internal consistency was 0.86. Also, in the study of Tayyebi, a significant relationship was obtained between this questionnaire and Interpersonal Reactivity Index (r = +0.33; P = 0.01) (15).

### 3.2.2. Affective Control Scale (ACS)

This scale is designed by Williams and Chambless (1992) and it has 42 items and four subscales (Anger, Depressed Mood, Anxiety and Positive Affect). To obtain the overall scale score, first convert the responses of reverse worded items, and then compute the average of all 42 responses. Cronbach’s Alpha in the study of Williams and Chambless (1992) was 0.94 in overall scale and varied from 0.72 to 0.91 in the subscales. Test-retest reliability for the total ACS score for two-week test-retest was 0.78 and in the subscales it varied from 0.66 to 0.77. Also, Construct Validity- overall scale discriminant validity with Marlow-Crowne Social Desirability Index obtained -0.17. Emotional Control Questionnaire convergent validity was -0.72 (P = 0.001) (16). According to the Cronbach's alpha of this study for the whole scale, the reliability coefficient of internal consistency was obtained to be 0.81. Also, in a study by Tayyebi, a significant relationship was obtained between this scale and Interpersonal Reactivity Index (r = -0.39 ; P = 0.01) (15).

### 3.3. Procedure

After selecting the research sample, the questionnaires were distributed to the subjects, and they were asked to complete the research instruments. The data was analyzed by multivariate analysis of variance (MANOVA) and LSD test.

## 4. Results

The results show that 32% of methamphetamine addicts, 21% of narcotic addicts, and 40% of non-addicts covered an age range of 20-29 years old. 68% of methamphetamine addicts, 79% of narcotic addicts, and 60% of non-addicts covered an age rang of 30-39 years old. Also, the results show that 68.6% of methamphetamine addicts, and 55.5% of narcotic addicts had precedent consecutive quitting. Table 1 shows the mean (and standard deviations) for self-regulation and affective control in methamphetamine addicts, narcotic addicts, and non-addicts. Table 2 shows that significance tests of self-regulation and affective control permit use of multivariate variance analysis. These results demonstrate that there is a significant difference between at least one of the dependent variables. Eta square shows that there is significant differences between three groups overall, and the rate of the differences is 32% in self-regulation and 26% in the affective control. According to Leven test and its insignificance for all of variables, condition of equality is considered in variances of groups. According to Box test, it is not significant for any of variables, and this condition of equality is considered as variance matrices (self-regulation: P = 0.26, F = 1.69, Box = 93.35; affective control: P = 0.13, F = 1.36, Box = 28.77).

**Table 1.** Mean and Std. Deviation of Self-regulation and Affective Control in the Three Groups

Self-Regulation and Affective Control	Methamphetamin Addicts, Mean ± SD	Narcotics Addicts, Mean ± SD	Non-Addicts, Mean ± SD
**Subscales of self-regulation**			
Receiving	27.75 ± 5.06	31.69 ± 5.83	31.92 ± 4.63
Evaluating	27.34 ± 4.58	27.89 ± 3.67	28.55 ± 3.88
Triggering	28.87 ± 3. 7	30.82 ± 3.08	31.12 ± 3.67
Searching	29.58 ± 4.55	31.23 ± 3.72	32.12 ± 3.04
Formulating	25.39 ± 5.7	28.74 ± 4.63	30.40 ± 4.33
Implementing	27.31 ± 4	30.12 ± 5.18	32.60 ± 4.08
Assessing	27.95 ± 3.98	29.10 ± 3.43	31.35 ± 2.30
**Total**	194.22 ± 18.67	209.62 ± 20.29	218.08 ± 14.56
**Subscales of affective control**			
Anger	29.21 ± 6.33	33.69 ± 6.03	34.87 ± 6.13
Positive	51.78 ± 9.33	57.84 ± 7.89	60.60 ± 7.61
Depression	28.85 ± 5.91	33.84 ± 5.67	35.52 ± 6.39
Anxiety	47.02 ± 10.38	58.23 ± 11.57	59.45 ± 9.71
**Total**	144.32 ± 15.69	158.92 ± 16. 8	160.25 ± 16.86

**Table 2.** The significance Test of MANOVA for Subscales of Self-regulation and Affective Control

	Value	F	Hypothesis Df	Error Df	Significance	Partial Eta Squared
**Self-regulation**						
Pillai's Trace	0.369	3.616	14	224	0.001	0.315
Wilks' Lambda	0.648	3.838	14	222	0.001	0.315
Hotelling' Trace	0.517	4.060	14	220	0.001	0.315
Roy's Largest Root	0.460	7.363	7	112	0.001	0.315
**Affective control**						
Pillai's Trace	0.274	4.566	8	230	0.001	0.259
Wilks' Lambda	0.730	4.860	8	228	0.001	0.259
Hotelling' Trace	0.365	5.151	8	226	0.001	0.259
Roy's Largest Root	0.349	10.039	8	115	0.001	0.259

Table 3 shows that there are significant differences between methamphetamine and narcotic addicts and non-addicts in the mean scores of Receiving (F = 8.24), Triggering (F = 4.9), Searching (F = 4.58), Formulating (F = 10.84), Implementing (F = 14.32), and Effectiveness (10.95) (P = 0.001). While the results show that there is no difference between the three groups in the mean scores of Evaluating (F = 0.89). Also, it can be concluded that there is significant difference between the three groups in the mean scores of Anger (F = 9.47), Positive affect (F = 11.9), Depression (F = 13.55), and Anxiety (F = 17) (P = 0.001).

**Table 3.** The MANOVA for the Mean Subscales of Self-Regulation and Affective Control

Scales	Dependent Variable	SS	Df	MS	F	Significance
**Self-regulation**	Receiving	444.681	2	222.341	8.241	0.001
	Evaluating	29.616	2	14.808	0.893	0.412
	Triggering	120.483	2	60.241	4.907	0.009
	Searching	134.617	2	67.309	4.581	0.012
	Formulating	528.508	2	264.254	10.845	0.001
	Implementing	556.155	2	283.077	14.32	0.001
	Assessing	241.199	2	120.6	10.95	0.001
**Affective control**	Anger	721.885	2	360.942	9.478	0.001
	Positive	1651.890	2	825.945	11.907	0.001
	Depression	977.026	2	488.513	13.55	0.001
	Anxiety	3802.793	2	1901.396	17.006	0.001

Abbreviations: SS; sum of squares, MS; mean square, F; f-ratio

According to Table 4, the results of LSD test for comparing the mean scores of subscales self-regulation demonstrate that methamphetamine addicts in comparison with narcotic addicts and methamphetamine addicts in comparison with non-addicts have lower mean scores in the Receiving, Triggering, Searching, and Formulating (P = 0.001); methamphetamine addicts in comparison with narcotic addicts; methamphetamine addicts in comparison with non-addicts and narcotic addicts in comparison with non-addicts have lower mean scores for Implementing (P = 0.01); also, methamphetamine addicts in comparison with non-addicts and narcotic addicts in comparison with non-addicts have lower mean scores in the Effectiveness (P = 0.001). Also, based on Table 5, results of LSD test for comparing the mean scores of subscales affective control depict that methamphetamine addicts in comparison with narcotic addicts; methamphetamine addicts in comparison with non-addicts have lower mean scores of Anger, Positive affect, Depression, and Anxiety (P = 0.001).

**Table 4.** The LSD Test for the Comparison Mean Scores Subscales of Self-Regulation

Group	2	3	Group	2	3
**Receiving**			**Formulating**		
Methamphetamine addicts	-3.93 (0.001)	-4.16 (0.000)	Methamphetamine addicts	-3.35(0.003)	5 (0.000)
Narcotic addicts		-0.23 (0.843)	Narcotic addicts	_	-1.65 (0.139)
Non-addicts	0.23(0.843)		Non-addicts	1.65(0.139)	
**Evaluating**			**Implementing**		
Methamphetamine addicts	-0.55 (0.543)	-1.2 (0.184)	Methamphetamine addicts	-2.81 (0.006)	-5.28 (0.000)
Narcotic addicts ****		-0.65 (0.478)	Narcotic addicts	_	-2.47 (0.015)
Non-addicts	0.65 (0.478)		Non-addicts	2.47 (0.015)	
**Triggering**			**Assessing**		
Methamphetamine addicts	-1.94 (0.015)	-2.24 (0.005)	Methamphetamine addicts	-1.15 (0.124)	-3.39 (0.000)
Narcotic addicts		-0.3 (0.700)	Narcotic addicts		-2.24 (0.003)
Non-addicts	0.3 (0.700)		Non-addicts	2.24 (0.003)	
**Searching**			**Total**		
Methamphetamine addicts	-1.64 (0.057)	-2.53 (0.003)	Methamphetamine addicts	-15.39 (0.000)	-23.85 (0.000)
Narcotic addicts		-0.89 (0.302)	Narcotic addicts		-8.45 (0.039)
Non-addicts	0.89 (0.302)		Non-addicts	8.45 (0.039)	

**Table 5.** The LSD Test for the Comparison Mean Scores Subscales of Affective Control

Group	2	3	Group	2	3
**Anger**			**Depression**		
Methamphetamine addicts	-4.47 (0.002)	-5.65 (0.000)	Methamphetamine addicts	-4.99 (0.000)	-6.67 (0.000)
Narcotic addicts	_	-1.18 (0.396)	Narcotic addicts	_	-1.67 (0.217)
Non-addicts	1.18 (0.396)	_	Non-addicts	1.67(0.217)	_
**Positive**			**Anxiety**		
Methamphetamine addicts	-6.06(0.001)	-8.81(0.000)	Methamphetamine addicts	-11.2(0.000)	-12.42(0.000)
Narcotic addicts	_	-2.75 (0.144)	Narcotic addicts	_	-1.21 (0.609)
Non-addicts	2.75 (0.144)	_	Non-addicts	-1.21 (0.609)	_
**Total**					
Methamphetamine addicts	-14.6 (0.000)	-15.93 (0.000)			
Narcotic addicts	_	-1.32 (0.721)			
Non-addicts	1.32 (0.721)	_			

## 5. Discussion

The aim of the current study was to compare self-regulation and affective control in methamphetamine and narcotic addicts and non-addicts. Results showed that there are significant differences between methamphetamine and narcotic addicts and non-addicts in self-regulation and affective control. In fact, the results showed that methamphetamine addicts in comparison with narcotics addicts and non-addicts; and narcotics addicts in comparison with non-addicts had lower self-regulation and affective control. These results are in line with the outcome of Sussman et al. (9), Salo et al. (17), Glassman et al. (10), Vik (12), Jensen-Campbell et al. (6), Gailiot et al. (3), Otten et al. (13), Cole et al. (2). From the viewpoint of the cognitive neuroscience researcher, deficits in regulation of cognition, emotion, or behavior, may depend on an individual’s ability of determination when adaptive control is required (18). Therefore, it can be very difficult for drug addicts to abstain from drug use (3). Also, according to numerous studies, substance abuse and their influence on brain occur in complicated structures such as the prefrontal cortex, and structures that serve motivation, or the limbic system, to aim at individual’s adaptive control and inhibit that substance addicts adapt and adjust effectively (3, 19). Therefore, substance addicts are involved in a recognition process of how to express and control their affects in the different situations, and defect in these skills influence different aspects of the personal life, interpersonal interaction, and mental and somatic health. Also, it is probable that substance addicts tend to act without thoughtfulness, which is an indicator of their low behavior control (9). Thus, as substance addicts cannot control thoughts, emotions, and behavior, if they encounter a defeat or anger they might avert conflicts and commit antisocial behaviors (6). In this way, they do not have successful interpersonal relationships (20). The findings presented in this study should be interpreted carefully. First, based on the self-reported scale and due to unconscious defense and prejudices in responses, the collected information brings about the possibility of information distortion. Second, addicts were selected only from one city and male addicts participated in this study; consequently, generalizations concerning self-regulation and affective control behaviors might be limited for other regions or the female addicts. Other limitations of the present study include the retrospective data collection and lack of follow-up information. Therefore, such studies should employ structured interviews and assess addiction symptoms and include an extended follow up period to track and examine relapse. Future research works should consider increasing the sample size and include both male and female addicts to test detailed hypotheses. Also, these results have important implications on pathology, prevention and treatment of methamphetamine and narcotic addicts.
